# Fragile Mentalizing: Lack of Behavioral and Neural Markers of Social Cognition in an Established Social Perspective Taking Task when Combined with Stress Induction

**DOI:** 10.1523/ENEURO.0084-24.2024

**Published:** 2024-11-26

**Authors:** Simrandeep Cheema, Calina Augustin, Martin Göttlich, Ulrike M. Krämer, Frederike Beyer

**Affiliations:** ^1^Department of Psychology, School of Biological and Behavioural Sciences, Queen Mary University of London, London E1 4NS, United Kingdom; ^2^University of Wolverhampton, Wolverhampton WV1 1LY, United Kingdom; ^3^Department of Neurology, University of Lübeck, Lübeck 23562, Germany; ^4^Institute of Medical Psychology, Center of Brain, Behavior and Metabolism, University of Lübeck, Lübeck 23562, Germany

**Keywords:** fMRI, mentalizing, perspective taking, stress

## Abstract

The growing field of social neuroscience is reliant on the development of robust, ecologically valid paradigms for simulating social interaction and measuring social cognition in highly controlled laboratory settings. Perspective taking is a key component of social cognition, and accordingly several paradigms aimed at measuring perspective taking exist. A relatively novel paradigm is the ball detection task, in which participants and a virtual agent form independent beliefs about the presence of a target stimulus behind an occluder. Previous studies have shown that incongruent trials (in which the participant's and the agent's beliefs differ) affect participant reaction times and elicit increased neural activity in the so-called mentalizing network. This paradigm has important advantages over previous ones, in that experimental conditions can be fully randomized, and ceiling effects are not found even for adult populations. Here, we combined this paradigm with a stress induction and a nonstressful control task. In an online study, we found no evidence of perspective taking at the behavioral level. Combining the task with functional magnetic resonance imaging, we found no evidence of perspective taking at the behavioral or neural level, even for the control condition. While this paradigm is reliable on its own, implementing it in the context of a task-switching paradigm appears to reduce participants’ focus on task-irrelevant perspective taking elements. Our findings highlight the fragility of existing social cognition paradigms and the need for reliable, simple, and ecologically valid measures of perspective taking.

## Significance Statement

Mentalizing, including the ability to represent another person's perspective and knowledge, is a core cognitive skill underlying social behavior. Studying intra- and interindividual differences in mentalizing, as well as neural correlates of mentalizing in different situations, for example, under stress, requires robust behavioral paradigms that can be utilized in neuroimaging settings. Our work highlights shortcomings of currently available paradigms, especially in the restricted context of neuroimaging, paving the way toward addressing these issues in the development of future tasks and measures.

## Introduction

In recent years, there has been a growing research interest in the impact of stress on social cognition and social behavior (von Dawans et al., 2021), but conflicting findings suggest important variability in the impact of stress on social cognition. For example, experimental manipulations of stress have been demonstrated to negatively affect social memory (Takahashi et al., 2004; Merz et al., 2010), impair spontaneous facial mimicry (Nitschke et al., 2020), and attenuate feelings of distress for others (Buruck et al., 2014). On the other hand, studies demonstrate that acute stress leads to increased performance on tasks measuring emotional empathy (Wolf et al., 2015) and increased BOLD activity from neural regions implicated in sharing of emotional states when viewing someone else under pain (Tomova et al., 2017). Other studies show that the effects of acute stress are moderated by gender, for example, Tomova et al. (2014) found that stress was linked to poor performance on self-other distinction tasks for male participants only.

Perspective taking is a core component of social cognition, as it is necessary for problem-solving and mitigating conflict when another person's beliefs and desires differ from our own (Rizkalla et al., 2008; Grant and Berry, 2011; Hoever et al., 2012; Gutenbrunner and Wagner, 2016). Importantly, such difficult social interactions are an important source of everyday stress (Almeida, 2005; Shahrestani et al., 2015), yet little is known about how acute stress impacts perspective taking.

Studying such effects requires robust, standardized experimental tasks which allow us to delineate intra- and interindividual differences in perspective taking. One recently developed and repeatedly validated task is the ball detection task (Kovács et al., 2010; El Kaddouri et al., 2020). In this task, participants watch videos where a ball rolls along a table and either stops behind an occluder or exits the scene in the presence of another agent. The agent then exits the scene, and the participant observes further movement of the ball either stopping behind the occluder or rolling out of the scene, before the agent returns. Thus, at this point the participant and the agent can hold congruent or incongruent beliefs about the ball's presence behind the occluder. The occluder then drops, and participants are instructed to indicate the presence of the ball as quickly as possible if revealed present, with the ball's presence manipulated randomly. Participants (P) respond faster if they believed that the ball was present (P+) than when they believed that the ball was absent (P−). The core measure, however, obtained from this task is the so-called Theory of Mind index (ToM-index): when participants hold the belief that the ball is absent behind the occluder, they are faster to detect the presence of the ball when the agent (A) believes the ball is present (P−A+) in comparison with when the agent does not (P−A−).

A core advantage of this task over another well-established measure of spontaneous perspective taking, the director's task (Dumontheil et al., 2010), is that task conditions are randomized on a trial-wise basis, and the task can be readministered to the same participant across different conditions. Furthermore, the task's focus on reaction times, rather than error rates, allows detection of small within-subject variations and avoids ceiling effects.

Combining this task with a stress induction, we recently showed that perspective taking under stress was enhanced for participants who reported regular physical exercise and for those with high resting heart rate variability (HRV), measured as beat-to-beat variance of heart rate (RMSSD; Kähkönen et al., 2021). These findings are in line with studies suggesting a positive effect of regular physical exercise on stress regulation (Huang et al., 2013) and positive links between resting HRV and emotion regulation (Thayer et al., 2009). However, this study was limited to female participants and behavioral indexes of perspective taking. Thus, the first aim of the current study was to replicate these effects in a larger, more representative sample by conducting an online study. Furthermore, we aimed to explore the neural correlates of this effect, in particular modulation of activity in the so-called mentalizing network.

Several studies have utilized the ball detection task to explore the neural correlates of implicit mentalizing (Kovács et al., 2014; Bardi et al., 2017; Nijhof et al., 2018). A consistent pattern has been observed whereby incongruent beliefs (P−A+/P+A−) elicit stronger activation in comparison with congruent (P−A−/P+A+) belief conditions during the belief formation phase in the right temporoparietal junction (rTPJ). Several meta-analyses (Van Overwalle, 2009; Denny et al., 2012; Schurz et al., 2014) support the recruitment of the rTPJ in behavioral paradigms that involve reasoning about another person's mental state. Therefore, activation of the rTPJ during the belief formation phase of the ball detection task reaffirms the notion that participants spontaneously mentalize about another's mental states when they are incongruent with the participant's own belief. Moreover, interindividual variability in neural response documented in the right TPJ during tracking of incongruent beliefs in the ball detection task has been related to early maltreatment or autism (Hudson et al., 2018; Nijhof et al., 2018). Boccadoro et al. (2019) carried out a meta-analysis on data pooled from three separate neuroimaging studies utilizing the ball detection task. A whole-brain analysis of the incongruent > congruent contrast showed neural activity from a large cluster encompassing the wider temporoparietal area that included the TPJ. Within this cluster, strongest peaks of activation were mapped within the middle temporal gyrus (rMTG), supramarginal gyrus (rSMG) and the lingual gyrus (rLING).

Thus, we conducted two experiments: firstly, an online experiment to study the impact of stress on the ToM-index in the ball detection task and relationships with exercise habits in a larger, mixed male and female sample; secondly, a study using functional magnetic resonance imaging (fMRI) to study the links between exercise habits, HRV stress, and neural correlates of perspective taking.

For the fMRI experiment, we predicted that stress induction would suppress activation across neural regions associated with mentalizing and this would be moderated by exercise habits and HRV. For both the online and fMRI study, we hypothesized that stress would lead to a reduction in the ToM-index, moderated by exercise habits.

## Materials and Methods

### Study 1: online study on the influence of exercise habits and acute stress on implicit mentalizing

#### Aims and hypothesis

The aim of the online study was to replicate the potential links between exercise habits and perspective taking under stress observed in Kähkönen et al. (2021) and to clarify whether this extends to a more diverse and larger sample tested through an online platform. We predicted that individuals with low levels of regular exercise would show a reduced ToM-index under stress. We further explored the role of self-reported emotion regulation skills in this effect.

##### Participants

To detect a similar effect to that in our previous study of *d* = 0.7–0.75 with a power of 80%, 46–52 participants would be required. Given the online setting of the task and the potential need to control for gender effects, we collected a sample of 95 healthy adults [mean (M) age, 20.8 years; standard deviation (SD), 4.4; 31 males and 64 females]. Participants were recruited through Prolific after passing the following prescreening check: fluency in English and being age 18 years or over. Participants were not excluded based on nationality or current residency. Participants were given monetary compensation for their participation (£7.50 per hour). All procedures were approved by the local ethics committee.

##### Implicit mentalizing task

**Figure 1.**
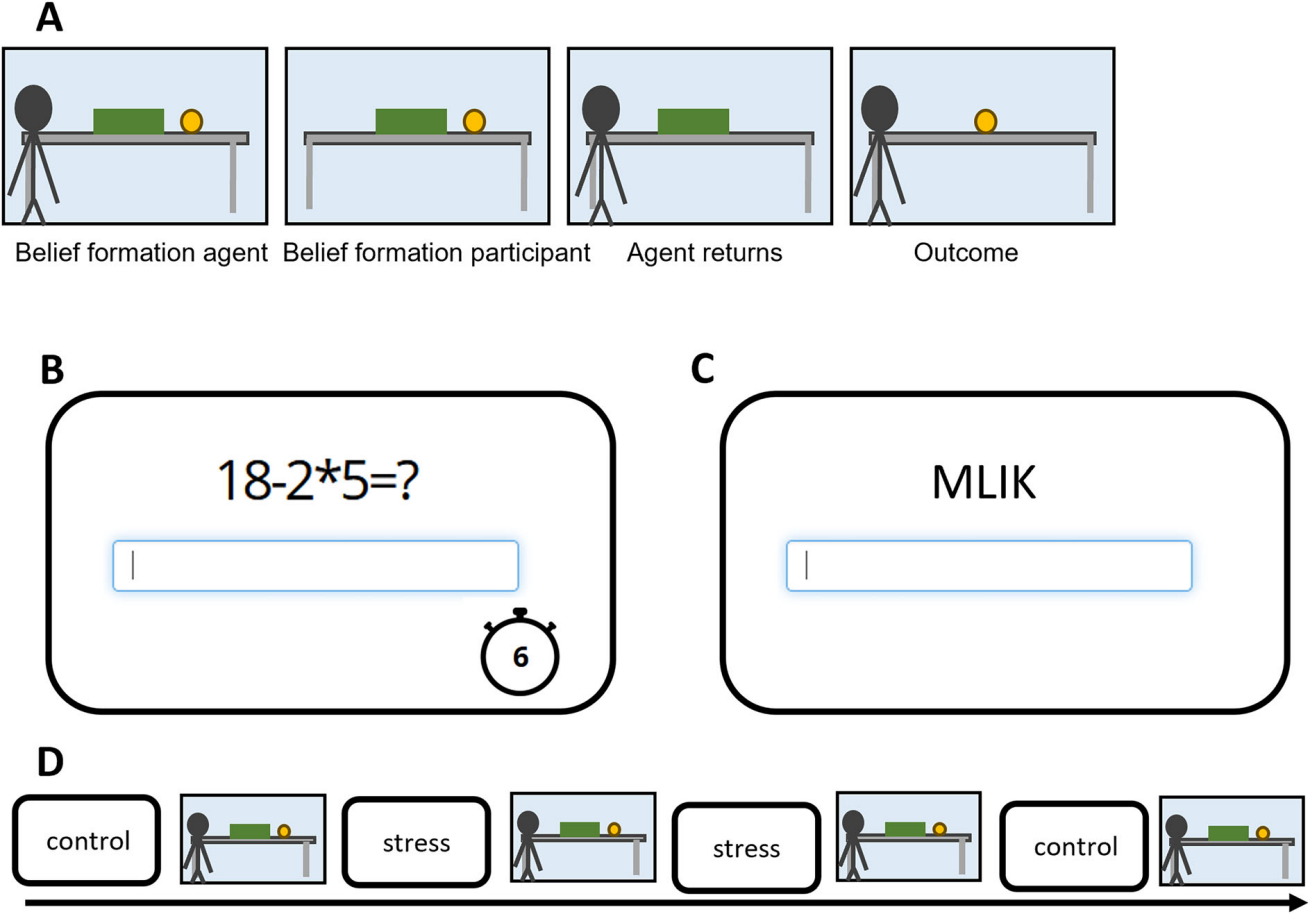
Task outline. ***A***, The trial outline of the ball detection task (El Kaddouri et al., 2020). An example trial of the stress induction task is shown in ***B*** and a trial of the control task in ***C***. ***D***, The order of tasks.

We used the ball detection task to assess implicit mentalizing (Kovács et al., 2010; El Kaddouri et al., 2020). This task was administered in an online setting through Gorilla (Cauldron Science), a professional tool for hosting behavioral experiments online using JavaScript. Across each trial (Fig. 1*A*), participants were instructed to watch a short movie clip where they saw a table, an occluder, and a virtual agent (Buzz Lightyear). The movie started with the agent placing a ball on a table, which then started rolling and either stopped behind the occluder or exited out of the screen. Subsequently, the agent would briefly exit the screen. Participants were instructed to press “v” on their keyboard to indicate when the agent left the screen to ensure they paid attention. During the agent's absence, the ball would move again, either stopping behind the occluder or outside the scene. The agent returned to the scene and subsequently the occluder fell. Participants were told to indicate with a keypress of “n” as quickly as possible if the ball was revealed once the occluder fell. The actual outcome of the trial, whether the ball was revealed as present or absent, was independent from the video shown and evenly split across trials. Based on the participant's (P) or agent's (A) belief before the occluder dropped (+, ball is present; −, ball is absent) and the outcome of the trial, eight conditions can be defined (P−A+, P+A+, P+A−, P−A ball absent; P−A+, P+A+, P+A−, P−A ball present).

Of particular interest is the comparison between P−A+ and P−A− trials in which the ball is revealed to be present as the occluder falls. The difference in reaction times between these conditions is referred to as the ToM-index, which has been used by previous empirical work as a behavioral index of implicit mentalizing. A positive ToM-index reflects that when participants believe the ball is not present behind the occluder, they are faster in their detection of the ball than when the agent believes the ball to be present (P−A+) in comparison with when the agent believes the ball to be absent (P−A−).

The ball detection task was conducted over four blocks with 20 trials each. Each block was preceded by either a stress induction (performing mental arithmetic under time pressure) or a nonstress control task (unscrambling easily solvable anagrams with no time limit) in the fixed order of nonstress→stress→stress→nonstress (Fig. 1*D*). Forty trials were displayed in a randomized order (five trials for each of the eight conditions) across the two blocks shown after the stress and nonstress induction, respectively. The ToM-index was computed separately for the stress and nonstress condition.

##### Stress induction: Montreal Imaging Stress Task

We adapted the Montreal Imaging Stress Task (MIST; Dedovic et al., 2005) that instructs participants to solve math equations under time pressure for an online setting. This task has been shown to reliably elicit increased heart rate and salivary cortisol, as well as the suppression of activity in brain regions associated with the major neuroendocrine systems implicated in the regulation of many body processes (Pruessner et al., 2008; Nagano-Saito et al., 2013).

Before completing the main task block including the ball detection task, participants were instructed to complete a short practice block of the MIST consisting of 10 trials. On each trial, participants were presented with a mathematical equation of medium difficulty to solve at their own pace and instructed to submit their answer in the text entry box provided. After submitting their answer in the text, appropriate feedback was displayed (“Correct!” or “Wrong!”). A central fixation cross displayed for 2 s preceded each trial. Once the practice block was terminated, the time taken to solve each question was averaged across all trials through the in-built Gorilla Java scripting function. This calculated variable was then used to determine the time available for participants to solve equations under time pressure (as indicated by a visible timer counting down to zero) across all the stress induction trials preceding the ball detection task. The duration of the visible timer allocated to each participant was determined as follows: fixed timer of 6 s if the output of calculated variable was <9 s, a fixed timer of 8 s if output of calculated variable was 9–12 s, a fixed timer of 10 s if the output of the calculated variable was between 12 and 15, and a fixed timer of 12 s if the calculated variable was >15 s, respectively.

Across the stress induction blocks preceding the ball detection task, the presentation of stimuli was identical to the practice block with the exception that participants were instructed to solve mathematical equations before a visible timer clocked down to zero (Fig. 1*B*). As outlined above, duration of timer was fixed at either 6, 8, 10, or 12 s depending on performance in the short practice block with no time pressure. After submitting their answer, a new screen was presented with feedback (“Correct!” or “Wrong!”). “Time Out” was displayed if the timer elapsed. Each block consisted of 10 trials.

##### Nonstress induction; anagrams

This task was created as a nonstressful control for the MIST described above. On each trial, participants were instructed to unscramble an anagram into a word corroborated from the English dictionary at their own pace (Fig. 1*C*). Anagrams consisted of three to four letter nouns or adjectives used in everyday life. Participants were instructed to type their answer in the text entry box provided using their keyboard. No feedback was provided after participants submitted their answer. A fixation cross displayed for 2 s preceded each trial. Each block consisted of 10 trials.

##### Questionnaires

The Emotional Regulation Questionnaire (ERQ; Gross and John, 2003) is a 10-item scale consisting of two subscales that measure the extent to which individuals regulate emotional experiences through expressive suppression and cognitive reappraisal. The former is an inhibitive response-focused strategy that aims to change the behavioral response after the emotional event is experienced, while the latter is a cognitive technique that aims to alter the meaning of the emotional experience while it is emerging. Participants provide responses on a five-point Likert scale from 1, “Does not describe me well,” to 5, “Describes very well.” The Interpersonal Reactivity Index (IRI; Davis, 1983) is a 28-item questionnaire consisting of four subscales that collectively measure individual differences in four aspects of dispositional empathy: perspective taking, fantasy, empathic concern, and personal distress. Participants provide responses on a five-point Likert scale from 1, “Strongly disagree,” to 5, “Strongly agree.” Finally, a Current and Lifetime Physical Activity Questionnaire

(CPAQ/LPAQ) was developed to quantify current exercise routine and engagement in exercise throughout early childhood, childhood, and adolescence for the present online study. The CPAQ consisted of two open-end questions instructing participants to state how many hours, on average, they spent exercising in a week and providing examples of exercises this would typically include. The LPAQ comprised of open- ended questions instructing participants to list a maximum of three examples of sport/exercise they engaged in and stating the duration (in years/months) and average hours spent per week for each listed exercise type. This was done across three separate time periods: early childhood (3–6 years), childhood (7–12 years), and adolescence (13–17 years).

Measurements of subjective levels of stress, in conjunction with ratings on other state emotions, to avoid biasing participants were collected at the end of the ball detection task. Participants were asked to rate separately how anxious, irritated, stressed, and relaxed they felt on a five-point Likert scale (from 1, “Strongly disagree” to 5, “Strongly agree”) when completing the stress and nonstress inductions, respectively.

##### Procedures

Presentation of all tasks and questionnaires were programmed in Gorilla (Cauldron Science), a professional tool for hosting behavioral experiments online using JavaScript. Participants were instructed to read an information sheet before deciding whether they would like to take part. After obtaining informed consent, participants were asked to complete a short practice session of all the tasks separately [in the order of ball detection (5 trials), mental arithmetic (10 trials), scrambled anagrams (5 trials)] to familiarize themselves with task instructions.

After completing the experimental tasks, participants completed the questionnaires online via Qualtrics.

#### Results

Out of the 95 participants tested, 36 participants were removed from statistical analysis due to poor performance on the ball detection task: (1) 10 or more false positives across all trials where ball was absent, i.e., they pressed a designated key to indicate that the ball was present once the occluder dropped when the ball was not, *N* = 12; (2) >1 miss in a critical condition used to calculate ToM-index on ball present trials, i.e., they failed to press designated key to indicate the ball is present in (P−A+) and (P−A−) trials, *N* = 32; and (c) if there were <3 trials for the critical conditions after excluding trials with individual RTs (reaction times) that were 3 SDs above or below the participant's mean RT, as well as those below 100 ms, *N* = 20. All analyses reported below were performed on the resulting sample of *N* = 59.

##### Manipulation checks

A Shapiro–Wilk test showed that self-reported ratings of subjective stress levels calculated for the stress and nonstress induction, respectively, did not significantly deviate from normality (*p* > 0.05). A paired samples *t* test was conducted to test whether the stress induction (psychosocial stressor; mental arithmetic task) elicited significantly higher levels of self-reported subjective stress in comparison with the control nonstress induction (easy scrambled anagrams). The psychosocial stressor task was found to significantly induce higher levels of self-reported stress (M, 3.3; SD = 0.71) in comparison with the nonstress control task (M, 0.45; SD, 0.82; *t*_(58)_ = −2.45; *p* = 0.001).

**Figure 2.**
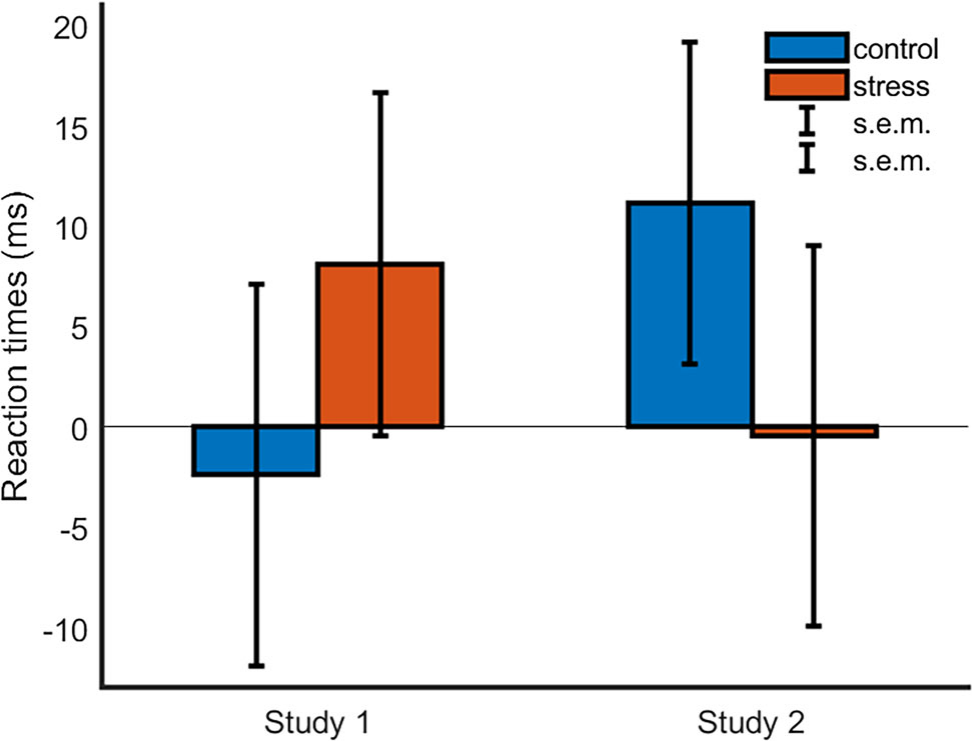
Behavioral data. The figure shows the ToM-indexes for the control and stress conditions for the first experiment (online study) and the second experiment (MRI study). Error bars show standard errors of the mean. None of the displayed measures were significantly different from 0 (*p* < 0.05 two-tailed).

To test for the presence of a significant ToM-index in the ball detection task, we ran one-sample *t* tests separately for the ToM-indexes of the control and stress conditions (Fig. 2). Both indexes were not significantly higher than zero (ToM-index^stress^; *t*_(58)_ = 0.96; *p* = 0.341; and ToM-index^control^; *t*_(58)_ = −0.25; *p* = 0.802).

In order to get a better idea of the robustness of these null effects, as well as to better account for both intra- and interindividual differences, we additionally analyzed reaction times using mixed linear models. This showed a significant effect for the factor of participant belief, with lower reaction times when participants believed the ball to be present. There was no effect of the agent's belief, nor a participant * agent interaction. Additionally including the factor of stress versus control condition showed a marginal effect of stress, but no interaction of stress with the belief factors (Table 1).

**Table 1.** Mixed linear model effects

	Coeff.	2.5% CI	97.5% CI	*t* value	*p* value
Model 1					
Participant belief	−0.015	−0.020	−0.009	−4.84	<0.001
Agent belief	−0.001	−0.007	0.005	−0.40	0.688
Participant * agent	<0.001	−0.005	0.007	−0.25	0.805
Model 2					
Participant belief	−0.014	−0.020	−0.009	−4.83	<0.001
Agent belief	−0.001	−0.007	0.005	−0.40	0.688
Stress	-<0.006	−0.012	<0.001	−1.95	0.051
Participant * agent	<0.001	−0.005	0.007	0.24	0.813
Participant * stress	−0.001	−0.007	0.005	−0.38	0.703
Agent * stress	−0.002	−0.008	0.004	−0.65	0.514
Participant * agent * stress	0.002	−0.004	0.008	0.78	0.436

The absence of an overall ToM-index in this dataset makes any interpretation of interaction effects with stress, exercise, and emotion regulation problematic. Nevertheless, we report the planned hypothesis checks for the sake of completion.

##### Analysis of main effects of stress on implicit mentalizing

To test the hypothesis that mentalizing will be suppressed under stress, we ran a paired *t* test between the ToM-index averaged across stress and control blocks, respectively. We found no significant difference (*t*_(58)_ = −0.81; *p* = 0.424.), with the ToM-index numerically higher in the stress (ToM-index^stress^; M, 8.14 ms; SD, 65.07 ms) relative to the nonstress condition (ToM-index^control^; M, −2.38 ms; SD, 72.47 ms).

##### Exploring effects of exercise and emotion regulation on social cognition

To test the influence of exercise and emotion regulation on implicit mentalizing, we ran two separate regression analyses using the ToM-index^stress^ and ToM-index^control^, respectively, as the criterion. In these linear regressions, the two subscales of the ERQ and current level of engagement in exercise taken from the CPAQ dichotomized as a binary variable (i.e., below 2.5, low; above 2.5, high) were entered as predictors. The linear regression model with ToM-index^stress^ entered as the criterion approached significance (*F*_(3, 55)_ = 2.65; *p* = 0.06); however all predictors were not significant (*p* > 0.05). The linear regression with ToM-index^control^ entered as the criterion returned as insignificant (*F*_(3, 55)_ = 1.07; *p* = 0.96), as were all predictors (*p* > 0.05; Table 2).

**Table 2.** Linear regression model testing effect of exercise and emotion regulation on implicit mentalizing (SEM, standard error of the mean)

	*ß*_stand_	SEM	*t*	*p*
ToM-index ^stress^				
ERQ cognitive reappraisal	−0.25	1.52	−1.93	0.10
ERQ expressive suppression	−0.25	2.13	−1.96	0.08
Exercise cutoff group	0.10	15.52	0.78	0.44
ToM-index ^stress^				
ERQ cognitive reappraisal	−0.38	2.17	−0.28	0.78
ERQ expressive suppression	−0.02	3.04	−0.14	0.89
Exercise cutoff group	0.07	22.15	0.50	0.62

#### Interim discussion

Overall, the most striking result was the lack of mentalizing (indicated by a nonsignificant ToM-index) observed in the nonstress control condition. It must be noted that over a third of our sample was removed due to poor performance on the ball detection task which may indicate poor attention and lack of engagement with the task. This may be because the task was conducted online, resulting in a higher dropout rate. We used strict inclusion criteria, only including data of participants who had high rates of passed attention checks and very low rates of missed target stimuli or false alarms. As such, we are confident only data of participants paying reasonably good attention to the videos was included. Nevertheless, it may be that subtle differences in reaction time underlying the ToM-index can only be detected in a strictly controlled laboratory setting where participant's attention can be controlled more closely and distractions minimized.

Thus, the follow-up study employed more stringent laboratory settings in addition to measuring the impact of stress on neural correlates of mentalizing alongside behavioral measures.

### Study 2: fMRI study on the influence of exercise habits, HRV, and stress on neural correlates of implicit mentalizing

#### Aims and hypothesis

The aim of the fMRI study was to investigate the links between exercise habits, HRV, and neural correlates of implicit mentalizing under stress. We predicted that a stress induction would suppress activation across neural regions associated with mentalizing and that this would be moderated by HRV and exercise habits.

##### Participants

Participants were recruited from the student population (undergraduate and postgraduate students) at the local university. The present study was advertised through flyers, word of mouth, and a circular email sent via relevant university-affiliated emailing lists. In order to detect a medium-sized effect in an independent *t* test for our a priori regions of interest, ∼30 participants per group were required. In total, 61 participants underwent fMRI scanning, but 1 participant did not complete all tasks. Participants received 10 euro/hour as compensation for participation. All procedures were approved by the local ethics committee.

Individuals who expressed interest in participating were first asked to complete a screening questionnaire about physical activity to recruit participants who fell within a low or high exercise group, respectively. The low exercise group consisted of individuals reporting that, on average, they spent ≤60 min per week on exercise in the last 6 months (*n* = 30; 26 females; M age, 22.8 years). The high exercise group consisted of individuals that reported spending an average estimate of 180 min or more on exercise per week in the last 6 months (*n* = 30; 22 females; M age, 23.3 years).

##### Implicit mentalizing task

The presentation of the ball detection task was the same as for the online study, with the exception that we split each 20-trial block into 2 blocks of 10 trials, to allow for more frequent stress induction. Thus, participants completed eight blocks in the following fixed order: C-C-S-S-S-S-C-C. This block order was chosen to ensure that participants experienced at least one block of the control condition prior to the stress induction (to reduce carry-over effects) while also controlling for the impact of time, by including the second half of the control condition in the end of the session. Trial timing for the ball detection task was as follows: intertrial interval (1.1–3.5 s); agent belief formation phase (9 s); participant belief formation phase (2.5 s); agent returns (0.5 s); and outcome phase (1 s). These timings are slightly longer than in previous studies, constrained by the playtime of the videos in belief formation phases.

##### Stress induction: MIST

**Figure 3.**
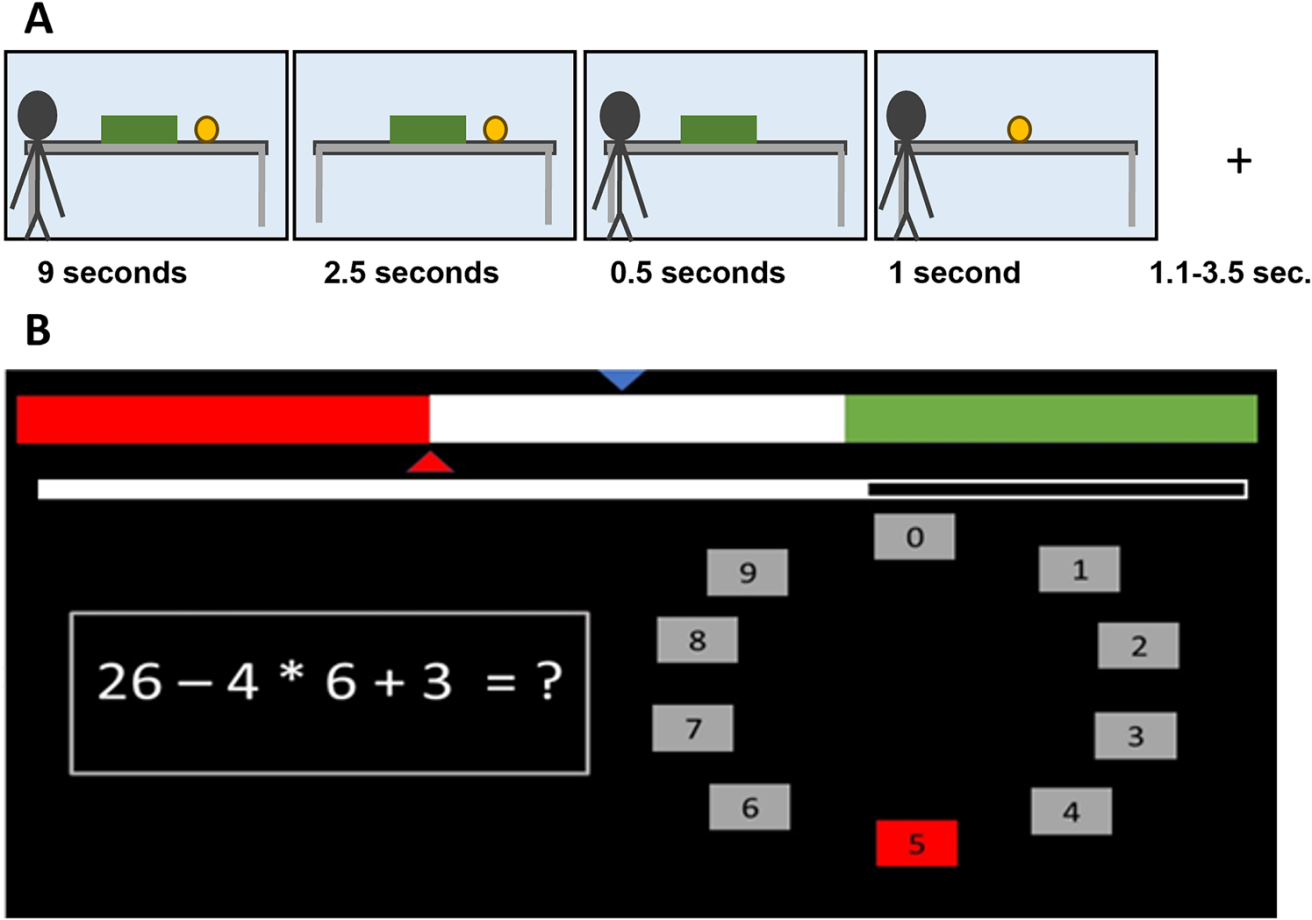
Task outline: ***A***, Timing of the ball detection task. ***B***, An example trial of the stress induction. Feedback bar is presented at the top of the screen, and the timer is below this.

As in Study 1, the MIST (Dedovic et al., 2005) was utilized to induce stress with a different response format and the additional element of a social evaluative component. In scanner settings across each trial, participants were instructed to solve the mathematical equation presented by moving a red marker around a circular dial, consisting of numbers 0–9, to submit their answer before the timer elapsed (Fig. 3). If participants submitted a response before the timer ran out, they received feedback translated into German (“Correct!” or “Wrong!”). Otherwise, “Time Out” was displayed if the timer elapsed. Each block lasted ∼2 min.

To elicit stress, the timer and difficulty of the math equations were adjusted in a trial-wise manner. For example, three consecutive correct responses resulted in a reduction of the time limit by 10% and an increase in the difficulty level for the next trial. Additionally, a socially evaluative element was incorporated, whereby participants were told that their performance would be assessed by the researcher to mark against an alleged group average. The group average was indicated by a blue arrow on a feedback bar positioned at the top of the screen. Participants were told to pay attention to the red arrow which moved from trial to trial to indicate their performance on the same feedback bar. Participants were told that placement of the red arrow in the red or green section indicated that they were performing below or above the group average, respectively. The difficulty level was always set to medium–easy difficulty from the onset of the task. This task was programmed through the Psychtoolbox extension in MATLAB (version R2018b).

Before entering the scanner, participants completed a practice block whereby they were told to solve equations at their own pace and no performance indicators (comparing participant's performance to a group average) were displayed. Participant's average response time was calculated from this practice session and then incorporated as a timer at the beginning of the first block of the stress induction. This practice session lasted ∼2 min.

##### Nonstress control induction: anagram

This task was created as a nonstressful control for the MIST described above. On each trial, participants were instructed to solve a scrambled word (in German) by selecting one of two answer options. Crucially, one answer was obviously wrong, as it included letters that were not present in the anagram, or it was not of the same length as the anagram. After submitting their response, the selected answer option was highlighted in blue. No feedback was provided. A fixation cross displayed for 1 s preceded each trial. Each block lasted ∼2 min.

##### Resting HRV

Five minutes of resting ECG were recorded after the MRI session. Raw ECG activity was collected with a BrainVision ExG Amp system which sampled at 2,000 Hz. Disposable Ag/Cl electrodes were attached in a lead III configuration to the participant's chest. MATLAB was used to transform the raw ECG signal into heartbeats per minute (bpm) using a peak detection formula based on the Pan–Tompkins algorithm programmed in MATLAB. HRV was calculated from the endmost 3 min of this period. In addition to visual inspecting and removing noisy epochs by plotting the ECG time series, a threshold percent-based algorithm was employed in MATLAB to detect implausible sudden jumps in the heart time series (i.e., heart rates that were 30% higher than the preceding heartbeat) and replaced values with NaN. A final value was calculated by taking the square root of differences between successive heartbeat intervals with the cleaned ECG data (time domain measure; RMSSD).

##### Questionnaires

The short version of the Cognitive Emotion Regulation Questionnaire (Garnefski and Kraaij, 2007) assesses the extent to which individuals use maladaptive or adaptive cognitive techniques to regulate negative emotional experiences. This questionnaire consists of 18 items which are rated on a five-point Likert scale from 0, “Almost never,” to 4, “Almost always.” The present study aggregated together individual subscales to create two variables representing overall use of adaptive (acceptance, positive refocusing, putting into perspective, refocus on planning, and positive reappraisal) and maladaptive (catastrophizing, other-blame, self-blame, and rumination) cognitive strategies, respectively, in line with Garnefski et al. (2001). The short version of the Big Five Inventory (Rammstedt and John, 2007) is a 10-item scale consisting of separate subscales that measure the following core dimensions of personality: agreeableness, neuroticism, conscientiousness, extraversion, and openness. Participants provide responses on a five-point Likert scale from 1, “Disagree strongly,” to 5, “Agree strongly.” Participants also completed the IRI (Davis, 1983).

Subjective levels of stress were assessed as in Study 1.

##### Procedures

Participants were asked to complete personality questionnaires online before attending the MRI session.

Once in the lab, before undergoing fMRI scanning, participants completed a short practice session to familiarize themselves with task instructions and practice trials across the different tasks that were administered as part of the fMRI protocol. This included five trials of the anagram task, five trials of the ball detection task, and a 2 min block of solving mental arithmetic at their own pace.

The main fMRI protocol consisted of four separate runs, each lasting ∼6 min. Each run involved two blocks of the task, in the following order: 1 min stress/anagram task and 10 trials of the ball detection task. Thus, each fMRI run included 2 min of the stress or anagram task and 20 trials of the ball detection task.

Across the two runs incorporating the stress and nonstress induction, respectively, all 40 trials of the ball detection task were completed in a randomized order.

The protocol for the fMRI study and data analysis plan (excluding exploratory analysis reported below) was preregistered on the Open Science Framework and can be accessed by https://osf.io/7ychp.

##### Neuroimaging data acquisition and preprocessing

fMRI data were acquired through the 3 T Siemens Magnetom Skyra MRI at the Center of Brain, Behavior and Metabolism with a 64-channel head coil. Across participants, functional images were collected over four runs (*N* = 478) with simultaneous multislice acquisition and the following parameters: repetition time, 1,000 ms; echo time, 30 ms; voxel size, 3 × 3 × 3 mm; and 56 transversal slices. At the end of the scanning session, a T1-weighted scan was obtained with voxel size 0.8 × 0.8 × 0.9 mm and 208 sagittal slices.

Preprocessing and statistical analysis of the fMRI data was conducted using SPM12, an open-source MATLAB toolbox (Wellcome Trust Centre for Neuroimaging). Firstly, functional images were corrected for differences in acquisition times through slice timing and were then realigned with fourth degree interpolation to account for head movements. Within-subject registration between the anatomical and functional images was implemented through rigid body transformations. The anatomical image was then spatially normalized into standard stereotactic MNI space using the unified segmentation procedure (version SPM12). Subsequently, the transformation parameters derived were applied to the functional images, which were based on the deformation field generated in the segmentation step. Finally, these images were spatially smoothed with an 8 mm Gaussian kernel.

##### First-level general linear model (GLM)

At the first-level subject analysis, a GLM was constructed for each participant.

This consisted of four regressors of interest: belief formation phase modeled separately for each trial type (congruent vs incongruent beliefs) and condition (stress vs control). A duration of 12 s was entered for all four regressors to reflect the period from the trial onset to the moment before the occluder fell. Regressors of no interest included modeling the outcome trial phase (when the ball is revealed as absent or present), the induction phase (anagram and MIST), as well as button responses for the detection of the ball and attention checks. Six regressors corresponding to realignment parameters were included to account for sources of noise introduced by head movements.

##### Preplanned statistical analyses

fMRI analyses were carried out on a priori defined ROI based on the findings reported by a recent meta-analysis (Boccadoro et al., 2019) that pooled data from three separate studies (Bardi et al., 2017; Hudson et al., 2018; Nijhof et al., 2018) employing the ball detection task to define neural correlates of implicit mentalizing. We constructed 5 mm spherical ROI around MNI peak coordinates (*x*, *y*, *z*) which are reported as follows: rTPJ, 57, −52, 34; rMTG, 60, −52, 1; rLING, 12, −73, 4; and rSMG, 54, −37, 46.

To test the regions involved in the implicit spontaneous tracking of beliefs, we conducted a paired *t* test with fixed effect analysis on first-level incongruent belief conditions (P−A+/P+A−) > congruent beliefs (P−A−/P+A+) contrast on the a priori defined ROI outlined above, separately for each condition (stress vs control).

To test the interaction between stress and implicit belief tracking, we conducted a paired *t* test with fixed effect analysis on first-level contrast of the difference in congruency between the stress and control condition on the a priori defined ROIs. This contrast was calculated as follows: [incongruent beliefs (P−A+/P+A−) > congruent beliefs (P−A−/P+A+) control] > [incongruent beliefs (P−A+/P+A−) > congruent beliefs (P−A−/P+A+) stress].

#### Results

##### Manipulation checks

A Shapiro–Wilk test showed that self-reported ratings of subjective stress levels calculated for the stress and nonstress induction, respectively, significantly deviated from normality (*p* < 0.05). A nonparametric Wilcoxon signed rank tests was conducted to test whether the stress induction elicited significantly higher levels of self-reported subjective stress in comparison with the control nonstress induction. The psychosocial stressor task was found to significantly induce higher levels of self- reported stress (*Median* = 3, *SD* = 0.83) in comparison with the nonstress control task (median, 0; SD, 0.75; *Z* = −2.45; *p* = 0.001).

##### Behavioral analysis

Out of the 61 participants tested, only 1 participant was removed from statistical analysis due to poor performance on the ball detection task. We used the same criteria for exclusion reported for the online study.

To test the hypothesis that mentalizing will be suppressed under stress, we ran a paired *t* test between the ToM-index averaged across stress and control blocks, respectively (Fig. 2). There was no significant difference (*t*_(59)_ = −1.01; *p* = 0.317) between the ToM-index in the stress (ToM-index^stress^; M, −0.45 ms; SD, 72.66 ms) relative to the nonstress condition (ToM-index^control^; M, 11.16 ms; SD, 61.44 ms). To test whether each respective ToM-index was significantly different from the null hypothesis, we ran one-sample *t* tests. Both indexes were not significantly higher than zero (ToM-index^stress^; *t*_(59)_ = −0.05; *p* = 0.962; and ToM-index^control^; *t*_(59)_ = 1.41; *p* = 0.165).

While numerically, the impact of stress on ToM-index appeared opposite to that found in Study 1 (Fig. 2), an ANOVA on ToM-index values with the within-subject factor condition (control vs stress) and the between-group factor study showed no significant effects (Table 3).

**Table 3.** ANOVA test statistics

	*F*	Degrees of freedom	Error degrees of freedom	*p* value
Condition	0.004	1	117	0.950
Study	0.076	1	117	0.783
Condition * study	1.622	1	117	0.205

We additionally analyzed the main behavioral effects of interest using mixed linear models, which showed a main effect for participant belief on reaction times, but no effect of agent belief nor an interaction of participant and agent belief. Thus, participants were faster when they believed the ball to be present, but this effect was not modulated by the agent's belief. Adding stress as a factor to the model did not change the results (Table 4).

**Table 4.** Mixed model effects for Study 2

	Coeff.	2.5% CI	97.5% CI	*t* value	*p* value
Model 1					
Participant belief	−0.014	−0.019	−0.009	−5.81	<0.001
Agent belief	−0.003	−0.008	0.002	−1.20	0.229
Participant * agent	<0.001	−0.005	0.004	−0.14	0.891
Model 2					
Participant belief	−0.014	−0.019	−0.009	−5.80	<0.001
Agent belief	−0.003	−0.008	0.002	−1.21	0.227
Stress	-<0.001	−0.005	0.005	−0.01	0.990
Participant * agent	-<0.001	−0.005	0.004	−0.13	0.894
Participant * stress	-<0.001	−0.006	0.004	−0.35	0.724
Agent * stress	<0.001	−0.005	0.005	0.07	0.945
Participant * agent * stress	−0.002	−0.007	0.002	−0.98	0.327

To test the influence of exercise and emotion regulation on spontaneous mentalizing, we ran two separate regression analyses using the ToM-index^stress^ and ToM-index^control^, respectively, as the criterion. In these linear regressions, the two subscales of the ERQ, HRV, and exercise group (i.e., high vs low) were entered as predictors. The linear regression model with ToM-index^stress^ entered as the criterion was nonsignificant (*F*_(4, 52)_ = 0.81; *p* = 0.52), as were all predictors (*p* > 0.05).

Similarly, the linear regression with ToM-index^control^ entered as the criterion returned as insignificant (*F*_(4, 52)_ = 0.84; *p* = 0.51), as were all predictors (*p* > 0.05; Table 5).

**Table 5.** Linear regression testing effect of exercise, physiological regulation, and emotion regulation on implicit mentalizing. (SEM, standard error of the mean)

	*ß*_stand_	SEM	*t*	*p*
ToM-index ^control^				
ERQ cognitive adaptive	−0.14	1.42	−1.93	0.36
ERQ cognitive maladaptive	−0.15	1.91	−1.96	0.37
Exercise group	0.09	15.89	0.64	0.53
HRV	−0.16	0.31	−1.12	0.27
ToM-index ^stress^				
ERQ cognitive adaptive	0.15	1.54	0.92	0.36
ERQ cognitive maladaptive	−0.05	2.07	−0.33	0.74
Exercise	−0.01	17.22	−0.10	0.92
HRV	0.16	0.33	1.15	0.26

##### Preplanned fMRI analyses

Besides the one participant excluded due to missing >50% of responses including both attention checks and detection of the ball in at least one MRI run, seven participants were excluded due to excessive head movements (>3 mm). Thus, the analyses conducted below were carried out on a final sample of *N* = 53.

**Figure 4.**
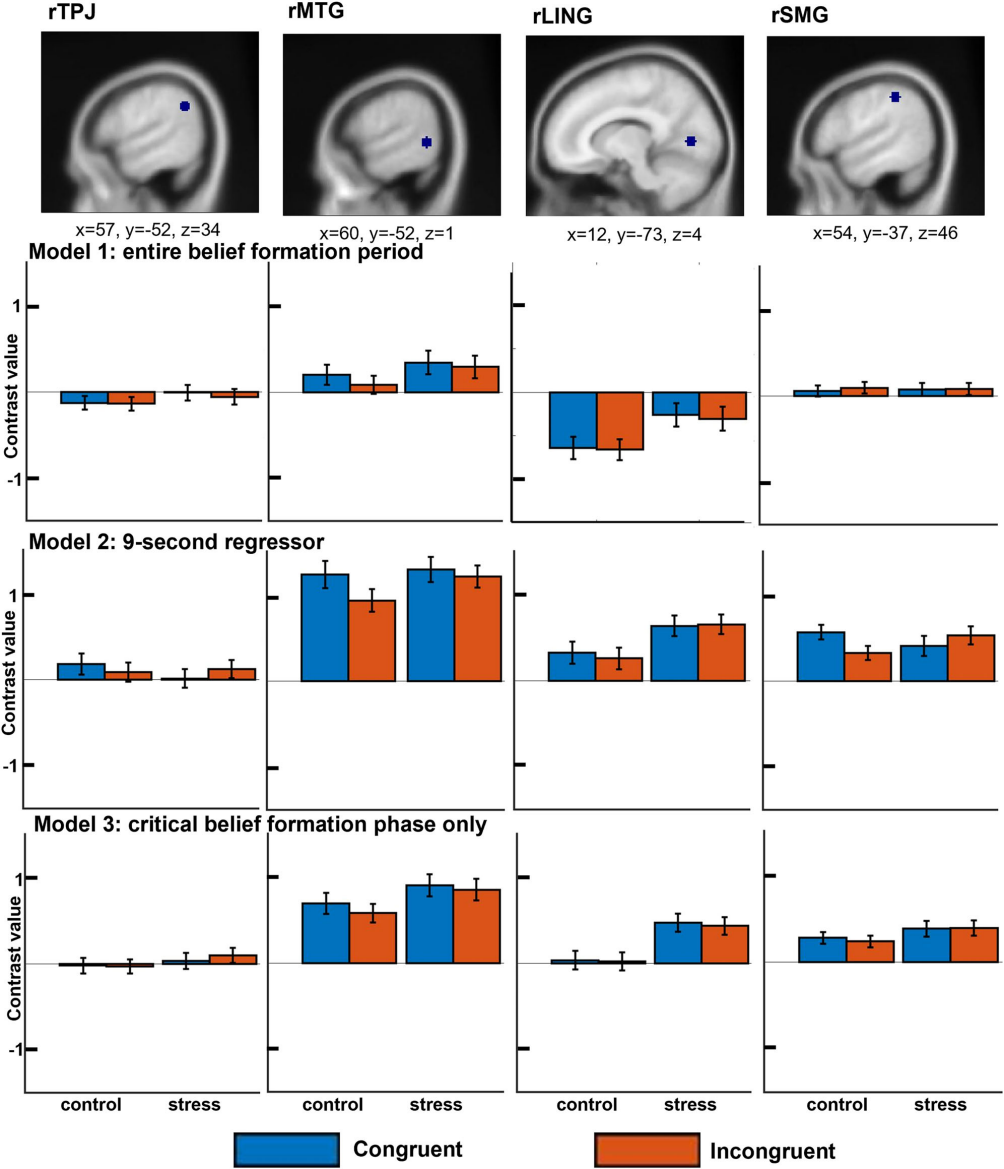
ROI data. The figure shows fMRI contrast values for the four regions of interest, separately for the three models run, and for the four experimental conditions (congruent and incongruent trials in the control and stress conditions).

For contrasts testing regions involved in the implicit spontaneous tracking of beliefs [incongruent belief conditions (P−A+/P+A−) > congruent beliefs (P−A−/P+A+)] for each condition separately (stress vs control), no voxels survived a corrected threshold of *p* = <0.05 (FWE; family-wise error) across the tests conducted. No voxels survived at an uncorrected *p* = <0.001 value threshold. The same test conducted over our predefined four ROIs found null results for all ROIs (Table 6; Fig. 4, Model 1), with Bayes factors showing moderate to strong evidence for the null hypothesis for all analyses except the comparison for rMTG in the control condition (which showed a trend opposite to the predicted effect, i.e., numerically higher contrast value for congruent trials).

**Table 6.** Statistical tests on regions of interest analyses

	*t* (p)			
	rTPJ	rMTG	rLING	rSMG
MODEL 1				
Control incongruent > congruent	−0.26(0.794) BF_(10) _= 0.155	−1.92 (0.060) BF_(10) _= 0.815	−0.45 (0.654) BF_(10) _= 0.165	−0.81 (0.424) BF_(10) _= 0.204
Stress incongruent > congruent	1.09 (0.280) BF_(10) _= 0.263	−1.13 (0.268) BF_(10) _= 0.273	−1.19 (0.240) BF_(10) _= 0.292	0.06 (0.948) BF_(10) _= 0.150
Stress × difference in congruency	−1.03 (0.308) BF_(10) _= 0.247	−0.77 (0.444) BF_(10) _= 0.198	0.49 (0.628) BF_(10) _= 0.168	−0.67 (0.508) BF_(10) _= 0.185

For the contrast testing the interaction between stress and implicit belief tracking ([incongruent beliefs (P−A+/P+A−) > congruent beliefs (P−A−/P+A+) control] > [incongruent beliefs (P−A+/P+A−) > congruent beliefs (P−A−/ P+A+) stress]), no voxels survived a threshold of *p* = <0.05 (FWE) corrected for multiple comparisons across the tests conducted. No voxels survived at an uncorrected *p* = <0.001 value threshold. The same test conducted over our predefined four ROIs also found null results (Table 6).

##### Exploratory fMRI analysis

As we did not find mentalizing activity in the belief formation phase of the incongruent beliefs (P−A+/P+A−) > congruent beliefs (P−A−/P+A+) contrast which has been consistently documented in previous literature for this task, we conducted post hoc exploratory analyses to explore whether this was due to issues with the GLM constructed at the first level. For this, we focused on the paired *t* test assessing neural activity associated with implicit mentalizing within the nonstress condition: [incongruent beliefs (P−A+/P+A−) > congruent beliefs (P−A−/P+A+) control]. Results for the stress condition are reported for the sake of completeness.

Analyses were run on the whole-brain level and on the predefined ROIs.

Our construction of the GLM at the first-level subject analysis level deviated from previous fMRI studies (Bardi et al., 2017; Hudson et al., 2018; Nijhof et al., 2018).

In previous studies, belief formation was modeled from the moment the agent placed the ball on the table to just after the agent reentered the scene after briefly leaving, with a fixed duration of 9 s regardless of video content. It may be that this shorter duration better encapsulates the period whereby participants would track discrepancies between their own and Buzz's belief, thus leading to better signal-to-noise ratio. In contrast, the present study used a longer duration for regressors of interest to model the entire 12 s from the beginning of the belief formation phase; consequently this may have reduced the sensitivity of our analyses. With this in mind, we conducted our first exploratory model as follows: four regressors for the belief formation phase modeled separately for each trial type (congruent vs incongruent beliefs) and condition (stress vs control) with a duration of 9 s and regressors of no interest modeling the outcome, induction phase and six motion parameters, respectively. When conducting second-level analyses with this GLM model, no voxels survived a threshold of *p* = <0.05 (FWE) corrected for multiple corrections for the incongruent (P−A+/P+A−) > congruent (P−A−/P+A+) contrast conducted for the stress and nonstress control condition, respectively. This was replicated for an uncorrected threshold of *p* = 0.001. Analyses restricted to ROIs also showed null results except for MTG and SMG which, in the control condition, showed the opposite effect as expected (higher activity in congruent trials; Table 7; Fig. 4, Model 2). Bayes factor analyses showed moderate support for the null hypothesis for nonsignificant results (Table 7).

**Table 7.** Statistical tests on post hoc exploratory analyses of different GLM models

	*t* (*p*)			
	rTPJ	rMTG	rLING	rSMG
Exploratory Model 1: 9 s regressor of interest				
Control incongruent > congruent	−0.91(0.368) BF_(10) _= 0.221	−2.97 (0.004) BF_(10)_ = 7.378	−0.90 (0.370) BF_(10) _= 0.220	−2.75 (0.008) BF_(10)_ = 4.378
Stress incongruent > congruent	1.03 (0.306) BF_(10) _= 0.248	−0.88 (0.386) BF_(10) _= 0.215	0.18 (0.860) BF_(10) _= 0.152	1.15 (0.842) BF_(10) _= 0.279
Exploratory Model 2: participant belief formation phase only				
Control incongruent > congruent	−0.24 (0.812) BF_(10) _= 0.154	−1.95 (0.056) BF_(10) _= 0.863	−0.33 (0.744) BF_(10) _= 0.158	−0.84 (0.402) BF_(10) _= 0.210
Stress incongruent > congruent	1.08 (0.282) BF_(10) _= 0.260	−1.11 (0.272) BF_(10) _= 0.268	−1.07 (0.290) BF_(10) _= 0.257	0.12 (0.908) BF_(10) _= 0.151

*t* coefficients and *p* values reported outside and inside the brackets, respectively.

In our second exploratory model, we decided to shorten the belief formation phase further by modeling the period just after the agent left, to the moment the occluder dropped. The first several seconds of the belief formation phase across the incongruent and congruent trials are identical, as incongruency only emerges after the agent has left the scene. Participant belief formation only happens toward the end of the movie once the ball moves in the agent's absence. Thus, excluding identical periods form the regressors of interest, and contrasting only the time period where differences between conditions emerge, should increase the sensitivity of our analyses. The agent belief formation phase of the trial was modeled as a separate regressor to avoid confounding the baseline. Additional regressors of no interest included the outcome, stress/control induction phase, and six motion parameters, respectively. When conducting second-level analyses with this GLM, no voxels survived a threshold of *p* = <0.05 (FWE) corrected for multiple corrections for the incongruent (P−A+/P+A−) > congruent (P−A−/P+A+) contrast conducted for the stress and nonstress control condition, respectively. This was replicated for an uncorrected threshold of *p* = 0.001. Analyses restricted to ROIs also showed null results (Table 7; Fig. 4, Model 3). Bayes factor analyses showed moderate support for the null hypothesis, except for MTG-control, which showed a *p* value approaching significance, and an inconclusive Bayes factor (Table 7).

## Discussion

The original aim of this study was to test the influence of exercise and HRV on the effects of stress on social cognition. We predicted that a stress induction would lead to a smaller ToM-index behaviorally and suppress activation across neural regions associated with mentalizing. Moreover, our second hypothesis predicted that HRV and exercise would moderate the effects of stress on implicit mentalizing.

However, across both studies, we found no evidence of implicit mentalizing in terms of behavioral or neural measures, in a relatively novel yet well-tested mentalizing task. Thus, our findings question the reliability of the task we used in eliciting perspective taking, when combined with more complex task setups, as discussed below.

Given the failure of detecting any perspective taking across the two studies, we will not discuss our original hypotheses of interactions between exercise, stress, and mentalizing.

### Lack of neural and behavioral evidence for mentalizing

Perhaps the most surprising finding was the absence of any activity in mentalizing areas for the incongruent > congruent contrast in the fMRI study, even for the control condition. This was true at the whole-brain level, even if using uncorrected *p* values, and for our a priori ROIs. Altogether, this contradicts a recent meta-analysis that pooled data from three separate studies (Bardi et al., 2017; Hudson et al., 2018; Nijhof et al., 2018) utilizing the ball detection task which found consistent activation of neural regions associated with mentalizing such as the rTPJ. This held true even when directly replicating the GLM conducted in previous studies (Bardi et al., 2017; Hudson et al., 2018; Nijhof et al., 2018) by shortening the duration of the incongruent and congruent regressors to 9 s and excluding button presses from the GLM. We found the same null results in our second exploratory model in which we reduced the duration of the incongruent and congruent regressors further by modeling the period just after the agent left to the moment the occluder dropped, to better encapsulate the onset of the participant's belief formation. Thus, across three variations of modeling the conditions of interest, we found no evidence of mentalizing-related activity in a whole-brain analysis nor within predefined ROIs, including the rTPJ.

Moreover, across both the online study and the fMRI study, we failed to find a significant ToM-index in either the control or stress conditions. This is in direct contrast to a good number of studies reliably detecting a ToM-index in both behavioral (Kovács et al., 2010; El Kaddouri et al., 2020) and neuroimaging (Bardi et al., 2017; Nijhof et al., 2018) contexts. It also directly contrasts with our own previous experiment using a very similar design of combining the ball detection task with a stress induction and control task (Kähkönen et al., 2021). In the following, we discuss differences in study design, which may explain this lack of mentalizing in our current experiments.

### Differences in methodology between the current and previous experiments

A key difference between our first experiment reported here and previous studies using the ball detection task is that our study was conducted online. The large proportion of participants who had to be excluded due to poor performance suggests that task engagement and attention were issues in this context. Lack of oversight may have resulted in participants engaging in other tasks besides the experiment or not paying attention to the instructions and the task itself. However, this issue was at least partially mitigated by excluding participants with failed attention checks from analysis. As evident from other recent online studies assessing social cognition (Goregliad Fjaellingsdal et al., 2023), stringent data exclusion criteria are necessary to ensure good data quality when testing online.

Another key difference between online and laboratory settings may be arousal levels. Increased arousal associated with being in a novel environment and potentially being observed by an experimenter may help achieve optimal arousal levels for simple, highly repetitive tasks such as the ball detection task used here. In line with this, in the online study, only the ToM-index for the stress condition approached significance, suggesting that at least, in this context, the stress induction did not have negative effects on spontaneous mentalizing.

These points highlight a core issue with the ball detection task, namely, that engaging in perspective taking is irrelevant for task performance. Even if participants are instructed to track the other agent's belief, which does not usually affect the ToM-index (Nijhof et al., 2016), the core task (responding quickly to the presence for the ball) does not require them to actively consider it during the outcome phase. Thus, participants can complete the task successfully while effectively disengaging from the trial for the first 12 s. Although they are required to press a button when the agent leaves the scene, this requires only minimal attention to the videos they are watching. Actively engaging with the videos and following the movements of the ball in each trial requires participants to voluntarily invest attentional efforts, which they may be more likely to do in a laboratory setting where they feel observed by an experimenter than when completing the task at home.

Both issues of controlling the study environment and achieving sufficiently high arousal levels to induce task engagement were expected to be resolved by the laboratory setting of the fMRI study. Indeed, previous studies successfully measured the ToM-index in fMRI studies (Bardi et al., 2017; Nijhof et al., 2018), and neural activity patterns associated with mentalizing in this task are considered robust (Boccadoro et al., 2019). Thus, other factors must have contributed to our failure to detect behavioral or neural indicators of perspective taking. We suggest that the task-switching requirements of our study design interfered with the attention dedicated to the ball detection task.

The fMRI protocol in the present study required the participant to complete repeated sessions of a stressor task, anagram task, and ball detection task in an alternating order. Converging empirical work demonstrates that task- switching has an adverse impact on performance for cognitive tasks and reaction times, respectively (Wylie and Allport, 2000; Kiesel et al., 2010; Worringer et al., 2019).

In our previous study (Kähkönen et al., 2021), we used a similar task-switching setup and found a significant ToM-index. However, subtle differences in the study designs may explain the discrepancy of these findings. In Kähkönen et al. (2021), the stressor task used required the participant to engage in repeated subtraction calculations as instructed and monitored by the experimenter. Participants received brief instructions on this at the beginning of this task, and the task was performed verbally and monitored by the experimenter. In this way, the experimenter eased the participant's cognitive load by directing the participant to carry out the task. The nonstress control task involved completing a simple word search on a paper with a pen, which required no prior instructions. Thus, participants were not asked to remember any task instructions for the stress or control tasks while carrying out the ball detection task. In contrast with this, our fMRI study involved a higher cognitive load as participants were required to remember different task instructions and response-button mapping across tasks. Participants practiced all three tasks outside the scanner and had to remember the instructions for each, before performing them inside the scanner. Thus, it may be that our fMRI design involved a high cognitive load, which meant that for the ball detection task, participants dedicated their attentional resources toward those elements of the task that were necessary for performance while ignoring task-irrelevant content of the videos.

In summary, our results suggest that while the ball detection task may be a robust measure of behavioral and neural indexes of perspective taking when implemented on its own in controlled laboratory settings, it can fail to measure mentalizing in less-controlled settings and in more complex study setups that increase the cognitive demand placed on participants. In fact, it may be the advantages of this task which set it apart from other measures of perspective taking that make the task easily fallible. Its focus on subtle reaction time differences means that this task can detect differences in perspective taking in adults, but it also means that this core measure is inherently noisy. It requires the repetition of many trials, which can bring about issues with attention and task engagement.

Furthermore, the fact that perspective taking is task irrelevant can be considered a strength of this paradigm. Other tasks explicitly requiring perspective taking suffer from ceiling effects in neurotypical adult populations (Brewer et al., 2017) or cannot be administered repeatedly and in randomized order with control conditions (Dumontheil et al., 2010). Some paradigms have attempted to address these issues by measuring reaction times in tasks involving conflicting perspectives (Samson et al., 2010). However, in these tasks, better performance is achieved by “not” engaging in perspective taking, meaning task performance is affected not only by an individual's inherent propensity to take another person's perspective into account but also by their cognitive control abilities.

The ball detection task addresses these issues by creating a setting in which perspective taking is not required for solving the task, avoiding ceiling effects and issues with task repetition. However, this simultaneously creates a situation where if the experimental setup becomes too demanding (or, in fact, too boring), it is straightforward for participants to ignore any material related to perspective taking and focus on nonsocial aspects of the task. This is in line with a recent study (Rothmaler and Grosse Wiesmann, 2023) using a task similar to the ball detection task, in which the authors failed to replicated influences of an observed agent's false belief on reaction times. As in the current study, in the study of Rothmaler and Grosse Wiesmann (2023), an additional cognitive demand was introduced, which the authors argue may have interfered with implicit, task-irrelevant perspective taking. Further highlighting the volatility of implicit mentalizing tasks, Haskaraca et al. (2023) recently failed to replicate false belief-dependent biases in another well-established perspective taking task.

Given the limitations of existing perspective taking tasks and the limitations of the ball detection task described here, the field is yet to develop robust perspective taking tasks that avoid ceiling effects and can be used in a range of study setups and experimental settings.

This will be of particular relevance to studies investigating neural measures of perspective taking. Given the constraints for the MRI environment, any combination of perspective taking tasks with other manipulations (such as stress induction) will create multitask setups, increasing cognitive load for the participant. Thus, simple, intuitive measures of perspective taking are needed which are sensitive to interindividual and intraindividual differences.

### Conclusion

In conclusion, the present study failed to detect behavioral and neural markers of perspective taking in two experiments, using a well-established perspective taking task. We suggest that the ball detection task works best when used on its own in controlled laboratory settings but has limited suitability for use in less-controlled environments or more complex task designs.

### Data availability

Anonymous data of reaction times and ROI analyses will be made available upon publication. Raw MRI data will not be published due to file size and anonymity restrictions.

## Synthesis

Reviewing Editor: Alexander Soutschek, Ludwig-Maximilians-Universitat Munchen Decisions are customarily a result of the Reviewing Editor and the peer reviewers coming together and discussing their recommendations until a consensus is reached. When revisions are invited, a fact-based synthesis statement explaining their decision and outlining what is needed to prepare a revision will be listed below. The following reviewer(s) agreed to reveal their identity: NONE.
